# High quality draft genome sequence of an extremely halophilic archaeon *Natrinema altunense* strain AJ2^T^

**DOI:** 10.1186/s40793-017-0237-3

**Published:** 2017-03-01

**Authors:** Hong Cheng, Ying-Yi Huo, Jing Hu, Xue-Wei Xu, Min Wu

**Affiliations:** 1 Laboratory of Marine Ecosystem and Biogeochemistry, Second Institute of Oceanography, State Oceanic Administration, Hangzhou, 310012 People’s Republic of China; 2 College of Life Sciences, Zhejiang University, Hangzhou, 310058 People’s Republic of China

**Keywords:** Halophilic archaea, High-altitude, Salt lake, Rhodopsin, Light-driven pumps

## Abstract

*Natrinema altunense* strain AJ2^T^, a halophilic archaeal strain, was isolated from a high-altitude (3884 m) salt lake in Xinjiang, China. This strain requires at least 1.7 M NaCl to grow and can grow anaerobically in the presence of nitrate. To understand the genetics underlying its extreme phenotype, we *de novo* assembled the entire genome sequence of AJ2^T^ (=CGMCC 1.3731^T^=JCM 12890^T^). We assembled 3,774,135 bp of a total of 4.4 Mb genome in only 20 contigs and noted its high GC content (64.6%). Subsequently we predicted the gene content and generated genome annotation to identify the relationship between the epigenetic characteristics and genomic features. The genome sequence contains 52 tRNA genes, 3 rRNA genes and 4,462 protein-coding genes, 3792 assigned as functional or hypothetical proteins in nr database. This Whole Genome Shotgun project was deposited in DDBJ/EMBL/GenBank under the accession JNCS00000000. We performed a Bayesian (Maximum-Likelihood) phylogenetic analysis using 16S rRNA sequence and obtained its relationship to other strains in the *Natrinema* and *Haloterrigena* genera. We also confirmed the ANI value between every two species of *Natrinema* and *Haloterrigena* genera. In conclusion, our analysis furthered our understanding of the extreme-environment adapted strain AJ2^T^ by characterizing its genome structure, gene content and phylogenetic placement. Our detailed case study will contribute to our overall understanding of why *Natrinema* strains can survive in such a high-altitude salt lake.

## Introduction

When the genus *Natrinema* was first described in 1998, it contained two species, *Natrinema pellirubrum* and *Natrinema pallidum* [1]. The genus *Natrinema* belongs to family *Halobacteriaceae*, phylum *Euryarchaeota*. Five more species of this genus were isolated and characterized since then, including *N. versiforme* [2], *N. altunense* [3], *N. gari* [4], *N. ejinorense* [5] and *N. salaciae* [6]. For now, the genomic sequences of all but *N. ejinorense* and *N. salaciae* in the genus *Natrinema* are publicly available on Genomes Online Database [7] and/or NCBI Genbank. Our lab first identified the *N. altunense* strain AJ2^T^ in 2005 in a salt lake [3]. Living cells in salt lake have made numerous adaptations to this special ecosystem, allowing them to flourish in a very harsh environment. To determine if the AJ2^T^ genome contains genes for adaptation to a particular set of environmental restrictions and supply a version of genome assembly in the database, we sequenced its whole genome in 2011 and published the whole genome sequence in the WGS database in May, 2014 as the first reported whole genome sequence of its species.

## Organism information

We isolated the strain AJ2^T^ from a water sample collected from the edge of Ayakekum salt lake (37°37′ N, 89°29′ E) in Altun Mountain (Altyn-Tagh) National Nature Reserve in Xinjiang, China (Table 1). This salt lake is cold and exposed to strong ultraviolet radiation throughout the year due to its high altitude. It also has high salinity and lacks the common organic nutrients for microorganisms [3].

**Table 1:** Classification and general features of *Natrinema altunense* AJ2^T^ [11]

MIGS ID	Property	Term	Evidence code^a^
	Current classification	Domain *Archaea* Phylum *Euryarchaeota* Class *Halobacteria* Order *Halobacteriales* Family *Halobacteriaceae* Genus *Natrinema* Species *Natrinema altunense* Type strain AJ2^T^=CGMCC 1.3731^T^=JCM 12890^T^	TAS [32] TAS [33, 34] TAS [33, 35] TAS [35, 36] TAS [37, 38] TAS [1] TAS [3]
	Gram stain	-	TAS [3]
	Cell shape	Rod	TAS [3]
	Motility	Motile	TAS [3]
	Sporulation	None	NAS [3]
	Temperature range	Not reported	TAS [3]
	Optimum temperature	Not reported	TAS [3]
	pH range; Optimum	5.5–9.0; 6.5–7.5	IDA
	Carbon source	Glucose, glycerol, maltose, glutamate, alanine, arginine, lysine, ornithine, acetate, fumarate, malate, propionate, pyruvate and succinate	TAS [3]
MIGS-6	Habitat	Salt lake	TAS [3]
MIGS-6.3	Salinity	Extremely halophilic. Growth requires 1.7 M NaCl (optimally 3.0–4.3 M) and grows in a wide range of 0.005–1.0 M MgCl_2_ (optimally 0.05–0.2 M).	TAS [3]
MIGS-22	Oxygen requirement	Aerobic. But the isolate can grow anaerobically in the presence of nitrate.	TAS [3]
MIGS-15	Biotic relationship	Free-living	NAS
MIGS-14	Pathogenicity	Not reported	
MIGS-4	Geographic location	Altun Mountain National Nature Reserve in Xinjiang, China	TAS [3]
MIGS-5	Sample collection	July, 2002	NAS
MIGS-4.1 MIGS-4.2	Latitude Longitude	37.62° N 89.48° E	TAS [3] TAS [3]
MIGS-4.4	Altitude	3884 m	TAS [3]

^a^Evidence codes - *IDA* Inferred from Direct Assay, *TAS* Traceable Author Statement (i.e., a direct report exists in the literature); *NAS* Non-traceable Author Statement (i.e., not directly observed for the living, isolated sample, but based on a generally accepted property for the species, or anecdotal evidence). These evidence codes are from the Gene Ontology project [39]

### Classification and features

**Fig. 1:**
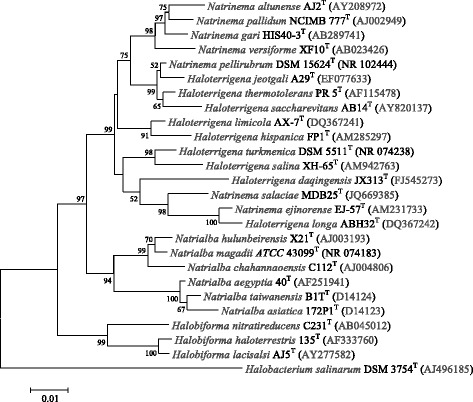
Phylogenetic tree highlighting the position of the Natrinema altunense strain AJ2^T^ relative to phylogenetically closely related type strains within the family *Halobacteriaceae*. These sequences were aligned on the SINA Online service [40] based on SILVA SSU/LSU databases. According to the best nucleotide substitution models found by the maximum-likelihood method in MEGA6 [41], the algorithm of the Jukes-Cantor model [42] was used to calculate the evolutionary distances in the neighbour-joining (NJ) method. Numbers at branch nodes refer to bootstrap values ≥ 50% (based on 1000 replicates). Halobacterium salinarum DSM 3754^T^ (AJ496185) was used as an out-group. Bar, 0.01 substitutions per nucleotide position

**Fig. 2:**
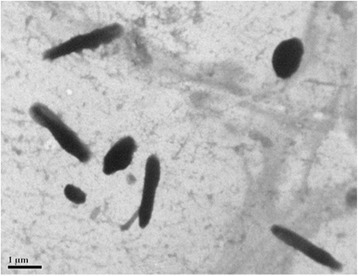
Transmission electron micrograph of cells of the strain AJ2^T^. Bar: 1 μm

*N. altunense* strain AJ2^T^ is an extremely halophilic archaea growing at 1.7–4.3 M NaCl and 0.005–1.0 M MgCl_2_. Colonies in the agar plate have a vivid orange or red colour. Cells are rod-shaped, but can become pleomorphic under unfavourable conditions as reported in 2005 [3]. The 16S rRNA gene sequence analysis was submitted to the EzTaxon-e service [8] and revealed 95.77–98.50% sequence similarity to members of the genus *Natrinema*. Strain AJ2^T^ exhibited the highest 16S rRNA gene sequence similarity with *N. gari* HIS40-3^T^ (98.50%). Phylogenetic analysis based on 16S rRNA gene sequences showed that strain AJ2^T^ clustered with most type strains of the genus *Natrinema* with a high bootstrap value (Fig. 1). The other three type strains, *N. pellirubrum* DSM 15624 ^T^, *N. salaciae* MDB25^T^ and *N. ejinorense* EJ-57^T^, were clustered with the genus *Haloterrigena*. *In the 16S rRNA gene trees* (Fig. 1) *and rpoB’ (RNA polymerase subunit B′) gene trees* [9]*, these three type strains of* genus *Natrinema* showed unclear taxonomic positions [10]. The mixture phylogenetic relationship *between these strains in the* *Natrinema* *and* *Haloterrigena* *genera were reported in 2003* [9]. This suggests that *Haloterrigena* maybe a later synonym (heterotypic) of genus *Natrinema*. The cell morphology and flagellum of *N. altunense* strain AJ2^T^ were examined using transmission electron microscopy (JEM-1230, JEOL). The cells of strain AJ2^T^ are straight and rods and have a diameter ranging 0.3–0.8 μm and length of 0.9–4.0 μm (Fig. 2). The cells are motile and their growth requires at least 1.7 M NaCl and 0.005–1 M MgCl_2_ (optimal 3.0–4.3 M NaCl and 0.05–0.2 M MgCl_2_). This strain is chemo-organotrophic and can anaerobically grow in the presence of nitrate. The strain had oxidase and catalase activity. The strain can reduce nitrate and nitrite and produce N_2_ gas. This strain can also hydrolyse gelatine and tweens 20, 40 and 80 as well as produce H_2_S from thiosulfate [3].

## Genome sequencing information

### Genome project history

We selected *N. altunense* AJ2^T^ for sequencing because its halophilic properties and high-altitude habitat may have caused interesting changes in its genome. Additionally, the five other members of genus *Natrinema* were sequenced and could be compared to our sequence (Table 2). This Whole Genome Shotgun project has been deposited in the DDBJ/EMBL/GenBank under the accession JNCS00000000. The version described in this paper is version JNCS00000000.1. Table 3 presents the project information and its association with MIGS version 2.0 compliance [11].

**Table 2:** The overall information of sequenced genomes about genus *Natrinema*

Species	Strain No.	Build year	Contig num.	GC %	Proteins	Total length (bp)	N50 (bp)	GOLD ID	INSDC	Assembly ID
Level: Contig										
*Natrinema pellirubrum*	DSM 15624^T^	1998	134	64.9	4176	4,264,455	83,437	Gi39311	AOIE00000000	GCA_000337635
*Natrinema pallidum*	DSM 3751^T^	1998	115	63.7	3844	3,915,814	88,603	Gi06160	AOII00000000.1	GCF_000337615.1
*Natrinema versiforme*	JCM 10478^T^	2000	72	64.0	4160	4,190,799	121,463	Gi0042913	AOID00000000.1	GCF_000337195.1
*Natrinema altunense*	**AJ2** ^**T**^	**2005**	**20**	**64.6**	**4462**	**3,774,135**	**425,349**	**Gi0074394**	JNCS00000000**.1**	**GCA_000731985.1**
*Natrinema altunense*	JCM 12890^T^	2005	52	64.5	3732	3,774,970	184,807	Gi06159	AOIK00000000.1	GCA_000337155.1
*Natrinema gari*	JCM 14663^T^	2008	88	63.7	4056	4,023,692	126,340	Gi0042887	AOIJ00000000.1	GCF_000337175.1
*Natrinema sp.*	J7-1	-	42	64.9	-	3,667,624	196,646	Gi17690	AJVG00000000.1	GCA_000493245.1
Level: Gapless Chromosome										
*Natrinema pellirubrum*	DSM 15624^T^	1998	1 Chromosome 1 Plsm: pNATPE01 1 Plsm: pNATPE02	64.9 57.0 58.3	3688 266 245	4,354,100	-	Gi05999 Gc0016535	CP003372.1 CP003373.1 CP003374.1	GCA_000230735.3
*Natrinema sp.*	J7-2	-	1 Chromosome 1 Plsm: pJ7-I	64.3 58.6	4302	3,697,626 95,989	-	Gi18911 Gc02274	CP003412.1 CP003413.1	GCA_000281695.1
Level: Non-Sequenced										
*Natrinema ejinorense*	JCM 13890^T^	2006	-	-	-	-	-	-	-	-
*Natrinema salaciae*	DSM 25055^T^	2012	-	-	-	-	-	-	-	-

The line highlighted with bold represent strain AJ2^T^

**Table 3:** Project information

MIGS ID	Property	Term
MIGS-31	Finishing quality	High-quality draft
MIGS-28	Libraries used	Paired-end 2000 bp library
MIGS-29	Sequencing platforms	GS FLX+ System
MIGS-31.2	Fold coverage	87× (2000 bp library)
MIGS-30	Assemblers	Newbler v. 2.5
MIGS-32	Gene calling method	Glimmer v3.02
	Locus Tag Genbank ID	ALTAJ2 JNCS00000000
	Genbank Date of Release	July 21, 2014
	GOLD ID	Gi0074394
	BIOPROJECT	PRJNA248700
MIGS 13	Source Material Identifier	CGMCC 1.3731^T^=JCM 12890^T^
	Project relevance	Ecosystem

### Growth conditions and genomic DNA preparation

*N. altunense* strain AJ2^T^ was aerobically cultivated at 37 °C for 3 days in modified CM medium, which contained the following (per liter distilled water): 7.5 g Casamino acid (Bacto), 10 g yeast extract (OXOID), 3 g trisodium citrate, 2 g KCl, 20 g MgSO_4_ · 7H_2_O and 200 g NaCl (pH 7.2). Genomic DNA was extracted according to the method described by Marmur & Doty [12]. The cells were suspended from 250 ml CM medium and washed once with 20% (w/v) NaCl solution. After extraction, the genomic DNA was dissolved in 1 ml of TE buffer. The quality and quantity of the genomic DNA was determined by 0.7% agarose gel electrophoresis with *λ*-Hind III digest and *λ*-EcoT14 I digest DNA marker (TaKaRa, Dalian, China) as well as by the DU800 spectrophotometer (Beckman Coulter, Inc.) with the nucleotide acid analysis method. The OD260/280 of genomic DNA was 1.92.

### Genome sequencing and assembly

The next-generation genome sequencing of *N. altunense* strain AJ2^T^ and quality control was performed using pyrosequencing technology on a GS FLX+ system (454 Life Sciences, Roche). One library with an insert size 2,000 bp was constructed and a total of 380 Mb clean data was obtained after filtering the adapter, artificial or low quality sequence. In other words we sequenced for a genome-wide average coverage of 87. A total of 630,866 reads were used for assembly and produced 20 contigs using the Newbler v.2.5 (454 Life Sciences, Roche). The average contig size was 188,706 bp and the largest contig size was 837,556 bp with the N50 size of 425,349 bp.

### Genome annotation

The tRNA genes of strain AJ2^T^ were identified using tRNAscan-SE 1.21 [13] with an archaeal model, and its rRNA genes were found via RNAmmer 1.2 Server [14]. Other ORFs were predicted using Glimmer3 [15]. The predicted ORFs were translated and analysed using the BLASTp program (BLAST 2.2.26+) against the non-redundant, Swiss-Prot [16], Pfam [17] and COG [18] databases. Only results with an e-value smaller than 1 × e^−5^ were kept. For cross-validation purposes, we annotated the genome with a RAST server online [19]. KAAS [20] was used to assign the predicted amino acids into the KEGG Pathway [21] with the BBH method. Genes with transmembrane helices were predicted using TMHMM Server v.2.0 [22]. We attempted to predict signal peptides using SignalP 4.1 Server [23], but because there were not enough experimentally confirmed signal peptides in the Uni-Prot database [23], the online server failed to provide the archaeal group model. The circular map of the genome was obtained using a local CGView application [24] with adjusted parameters (−size medium -title ‘AJ2^T^’ -draw_divider_rings T -gene_decoration arc -linear circular). We uploaded the whole genome sequences in FASTA files and calculated the ANI value between every two genome sequences within the genus *Natrinema* and *Haloterrigena* on the EzGenome online server [25, 26]. Genome accession numbers for all five published *Natrinema* and *Haloterrigena* strains are listed as follows: *N. altunense* AJ2 (JNCS00000000); *N. versiforme* JCM 10478 (AOID00000000); *N. pallidum* DSM 3751 (AOII00000000); *N. pellirubrum* DSM 15624 (CP003372); *N. gari* JCM 14663 (AOIJ00000000); *H. thermotolerans* DSM 11522 (AOIR00000000); *H. salina* JCM 13891 (AOIS00000000); *H. limicola* JCM 13563 (AOIT00000000); *H. turkmenica* DSM 5511 (CP001860); and *H. jeotgali* A29 (JDTG00000000). Unless otherwise specified, we used default parameters for all software.

## Genome properties

**Fig. 3:**
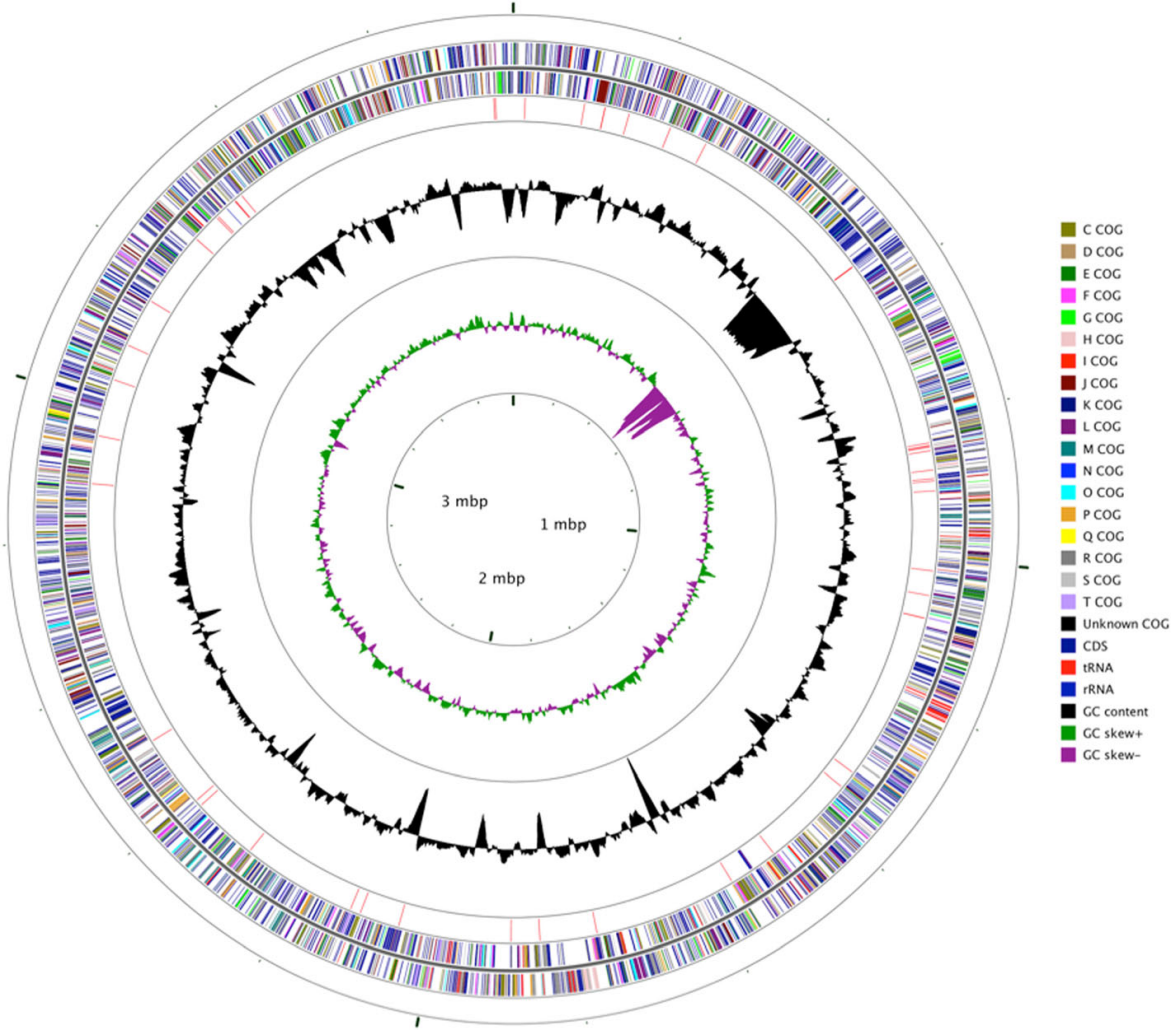
Graphical circular map of the genome of *N. altunense* AJ2^T^. Labelling from outside to the center: circle 1, CDSs on the forward strand (coloured by COG categories); circle 2, CDSs on the reverse strand (coloured by COG categories); circle 3, RNA genes (tRNAs red and rRNAs blue); circle 4, G + C content (peaks out/inside the circle indicate values higher or lower than the average G + C content 64.65%, respectively); circle 5, GC skew (calculated as (G-C)/(G + C) using a window size of 10000 and step of 100, green/purple peaks out/inside the circle indicates values higher or lower than average GC skew value (−0.0047), respectively); and circle 6, Genome size (Mbp)

This high-quality draft genome sequence of *N. altunense* AJ2^T^ revealed a genome size of 3,774,135 bp (all 20 contigs length, 64.56% GC content). We predicted 4517 genes; 4462 are protein-coding sequences. A total of 3792 protein-coding genes (83.95%) were assigned to a putative function or as hypothetical proteins. We also found 52 tRNA genes (removed 1 Pseudo tRNA) and 3 rRNA genes (one 23 S rRNA, one 16 S rRNA and one 5 S rRNA). We assigned 1929 protein-coding genes (42.71%) to Pfam domains and categorized 2255 (49.92%) protein-coding genes into COGs functional groups (Table 4 and Fig. 3). This genome has a gene content redundancy of 36.11%, and there are 1631 protein coding genes belonging to 540 paralog clusters. The genomic ANI values within the *Natrinema* and *Haloterrigena* genera are listed in Table 5. In the Richter & Rosselló-Móra report, the proposed ANI cut-off for the species boundary is at 95 ~ 96% [25]. According to our calculation data, the ANI values between any two species of *Natrinema* with published genome sequences were lower than 93.2% and this value was observed between strains AJ2^T^ and *Natrinema pallidum* DSM 3751 ^T^. We can also easily observe that *N. pellirubrum* show higher ANI values (>95%) with *H. thermotolerans* DSM 11522 ^T^ (95.4%) and *H. jeotgali* A29^T^ (95.2%). These data are also identical to the phylogenetic distance in the 16S rRNA maximum-likelihood tree (Fig. 1). In the tree, the other two strains *N. salaciae* MDB25^T^ and *N. ejinorense* EJ-57^T^, which are in the same clade as genus *Haloterrigena*, lack of genome information for considering their ANI values in this study.

**Table 4:** Number of genes associated with general COG functional categories

Code	Value	% age	Description
J	163	5.98	Translation, ribosomal structure and biogenesis
A	1	0.04	RNA processing and modification
K	155	5.69	Transcription
L	135	4.95	Replication, recombination and repair
B	3	0.11	Chromatin structure and dynamics
D	26	0.95	Cell cycle control, Cell division, chromosome partitioning
V	39	1.43	Defense mechanisms
T	120	4.40	Signal transduction mechanisms
M	97	3.56	Cell wall/membrane biogenesis
N	19	0.70	Cell motility
U	26	0.95	Intracellular trafficking and secretion
O	123	4.51	Posttranslational modification, protein turnover, chaperones
C	188	6.90	Energy production and conversion
G	98	3.60	Carbohydrate transport and metabolism
E	225	8.26	Amino acid transport and metabolism
F	74	2.72	Nucleotide transport and metabolism
H	147	5.39	Coenzyme transport and metabolism
I	112	4.11	Lipid transport and metabolism
P	183	6.72	Inorganic ion transport and metabolism
Q	48	1.76	Secondary metabolites biosynthesis, transport and catabolism
R	471	17.28	General function prediction only
S	272	9.98	Function unknown
-	2073	46.46	Not in COGs

The total is based on the total number of protein coding genes in the genome

**Table 5:** ANI values between genome pairs within genus *Natrinema* and *Haloterrigena*

→	*N. altunense*	*N. versiforme*	*N. pallidum*	*N. pellirubrum*	*N. gari*	*H. thermotolerans*	*H. salina*	*H. limicola*	*H. turkmenica*	*H. jeotgali*
*N. altunense*	-	83.50%	93.22%	82.89%	92.75%	82.82%	79.84%	80.99%	79.94%	82.89%
*N. versiforme*	83.09%	-	82.96%	82.40%	82.92%	82.31%	80.41%	80.90%	80.20%	82.37%
*N. pallidum*	93.21%	82.75%	-	82.75%	91.65%	82.74%	79.78%	80.93%	79.70%	82.79%
*N. pellirubrum*	83.00%	82.36%	82.98%	-	82.69%	95.39%	80.00%	80.83%	80.20%	95.16%
*N. gari*	92.82%	82.59%	91.89%	82.39%	-	82.48%	79.63%	80.69%	79.75%	82.65%
*H. thermotolerans*	82.65%	82.29%	82.35%	95.49%	82.33%	-	80.03%	80.64%	80.30%	97.36%
*H. salina*	79.45%	79.87%	79.38%	79.91%	79.13%	79.91%	-	78.77%	90.60%	79.96%
*H. limicola*	80.98%	81.15%	80.89%	81.12%	80.81%	80.88%	79.43%	-	79.49%	81.34%
*H. turkmenica*	79.69%	80.25%	79.42%	80.22%	79.41%	80.12%	91.13%	79.14%	-	80.29%
*H. jeotgali*	82.56%	82.42%	82.79%	95.14%	82.43%	97.41%	80.11%	80.81%	80.35%	-

The calculated genomic sequence used: *N. altunense* AJ2 (JNCS00000000); *N. versiforme* JCM 10478 (AOID00000000); *N. pallidum* DSM 3751 (AOII00000000); *N. pellirubrum* DSM 15624 (CP003372); *N. gari* JCM 14663 (AOIJ00000000); *H. thermotolerans* DSM 11522 (AOIR00000000); *H. salina* JCM 13891 (AOIS00000000); *H. limicola* JCM 13563 (AOIT00000000); *H. turkmenica* DSM 5511 (CP001860); *H. jeotgali* A29 (JDTG00000000)

## Insights from the genome sequence

We compared all sequenced strains in the genus *Natrinema* with strain AJ2^T^ according to the contig numbers, G + C content, predicted protein numbers, total length and N50, which are listed below (Table 6). The other relevant genomic features were listed in Table 7. According to the chemotaxonomic information and characteristic features of strain AJ2^T^ that was mentioned before, the strain contains a flagellin domain protein in its genomic features to support cell motility. It also has DNA repair systems for protecting the stability of its genome from potential damage caused by UV radiation. Additionally, the energy converting system and light-driven pumps are introduced below.

**Table 6:** Genome statistics

Attribute	Value	% of total
Genome Size (bp)	3,774,135	-
DNA coding (bp)	3,316,088	87.86
DNA G + C (bp)	2,436,432	64.56
DNA scaffolds	20	-
Total genes	4517	-
Protein-coding genes	4462	98.78
RNA genes	55	1.22
Pseudo genes	-	-
Genes in internal clusters	540	11.95
Genes with function prediction	2215	49.04
Genes assigned to COGs	2255	49.92
Genes with Pfam domains	1929	42.71
Genes with signal peptides	-	-
Genes with transmembrane helices	879	19.46
CRISPR repeats	-	-

**Table 7:** The relevance characteristics with genomic features annotation

Relevant characteristics	ID	Contig	Position	Strand	Annotation
Tween degradation	AJ2_rast_231	1	198927:200015	+	esterase/lipase
	AJ2_rast_522	1	476323:477450	−	putative esterase
Thiosulfate degradation	AJ2_rast_3344	11	30688:31554	−	thiosulfate sulfurtransferase2C rhodanese (EC 2.8.1.1)
	AJ2_rast_3346	11	31834:32646	+	thiosulfate sulfurtransferase (EC:2.8.1.1)
H_2_O_2_ degradation	AJ2_rast_1204	2	332019:334157	+	catalase (EC 1.11.1.6)/Peroxidase (EC 1.11.1.7)
	AJ2_rast_3782	16	4816:5718	−	catalase (EC:1.11.1.6)
Nitrous oxide reductase	AJ2_rast_1974	4	104337:105296	−	nitrous oxide reductase maturation transmembrane protein NosY
	AJ2_rast_2203	4	324166:325008	+	nitrous oxide reductase maturation transmembrane protein NosY
	AJ2_rast_3199	10	18205:19152	−	nitrous oxide reductase maturation transmembrane protein NosY
	AJ2_rast_3201	10	20059:21438	−	nitrous oxide reductase maturation protein NosD
	AJ2_rast_3203	10	22285:24204	−	nitrous-oxide reductase (EC 1.7.99.6)
Motility	AJ2_rast_1043	2	171217:173058	+	flagella-related protein FlaI
	AJ2_rast_1170	2	296018:296341	+	chemotaxis regulator CheY
	AJ2_rast_1825	3	392764:394281	+	conserved flagella cluster protein
	AJ2_rast_2104	4	230767:231792	−	signal peptidase2C type IV - prepilin/preflagellin
DNA repair	AJ2_rast_1703	3	284669:285400	+	DNA repair and recombination protein RadB
	AJ2_rast_2261	4	382800:384209	−	single-stranded-DNA-specific exonuclease RecJ (EC 3.1.-.-)
	AJ2_rast_2296	4	413891:414922	+	DNA repair and recombination protein RadA
	AJ2_rast_2880	8	16958:18862	+	RecJ like exonuclease

### Light-driven pumps

The strict living environment and lack of nutritious carbon/nitrogen sources cause diversification of metabolic pathway strain AJ2^T^ and similar halophilic archaea, as well as for haloarchaea, with more resources. Strain AJ2^T^ might use sunlight to produce ATP. We predicted the existence of two light-energy-converting system genes in the AJ2^T^ genome, namely *bop* and *hop.* The two encode homologous proteins bacteriorhodopsin and halorhodopsin, respectively. Bacteriorhodopsin and halorhodopsin share 36% of the amino acid residues in the transmembrane part and 19% in the surface connecting loops [27].

Bacteriorhodopsin is an integral membrane protein, called purple membrane, located in the archaea cell membrane, and it acts as a light-driven proton pump. It is mainly found in the *Halobacteriaceae* family [28, 29]. It captures and uses light energy to move protons out of the cell membrane, resulting in a proton electrochemical gradient. Subsequently, the gradient is converted into chemical energy through ATP synthesis or is used to fuel flagellar motility and other energy requiring processes [30]. We obtained the complete *bop* gene (AY279548, JQ406920, and AFB77278) in the strain AJ2^T^ by the LPA method. We then successfully expressed the AJ2^T^ bacteriorhodopsin protein in *E.coli* BL21 with recombinant pET28a plasmid. This result indicates that the prediction of the *bop* gene is correct. Halorhodopsin is a light-activated chloride pump that is also found in archaea. It utilizes light to transfer the chloride ions into the cytoplasm and increase the electrochemical potential of the proton gradient [31]. This gene is extremely important for salty environment tolerance and, by reporting the existence of a *hop* gene in the *N. altunense* strain AJ2^T^, we shed light on the potential mechanism of its adaptation to high salinity.

Bacteriorhodopsin, halorhodopsin and several related bacterio-opsin activator HTH domain proteins were also found in the other sequenced type strains *N. pellirubrum*, *N. pallidum*, *N. gari* and strain *Natrinema* sp. J7-2 (listed in Table 8). As the haloarchaea species of the genus *Natrinema* *typically live in similar environment*, this type of bacteriorhodopsin/halorhodopsin-based phototrophy can help them adapt to extremely hypersaline and oligotrophic niches.

**Table 8:** Bacteriorhodopsin and halorhodopsin in the genomes of genus *Natrinema*

Species	Strain	Bacteriorhodopsin		Halorhodopsin	
		Size/aa	Accession No.	Size/aa	Accession No.
*N. pellirubrum*	DSM 15624^T^	223	WP_006180343	281	WP_006179856
*N. pallidum*	DSM 3751^T^	223	WP_006186147	282	WP_006185564
*N. altunense* ^a^	AJ2^T^	223	AFB77278	285	KY435894
*N. gari*	JCM 14663^T^	223	WP_008455435	282	WP_008453746
*Natrinema sp.*	J7-2	223	YP_006542121	278	YP_006540994

^a^This data line represents the closest output obtained using BLASTp program against the nr database. These two genes are on contig 1 (position:629096–629767, forward strand) and contig 3 (position:389528–390385, forward strand) of the genome of strain AJ2^T^, respectively

## Conclusions

The genome of strain AJ2^T^ did not have the longest length in the sequenced strains of *Natrinema* *,* but it had most predicted proteins. Meanwhile, the assembled result in the strain AJ2^T^ had the lowest contig numbers and largest N50 length. This indicated the larger size of the library (2000 bp library) and the longer read length (up to 1000 bp with an average read length 603 bp) may significantly improve the assembling quality.

Our genomic analysis of strain AJ2^T^ shed light on its ability to survive in the Ayakekum salt lake of Altun Mountain National Nature Reserve in Xinjiang, China. This lake is regarded as a relatively extreme environment with low nutrient levels, a cool temperature, strong sunlight and high-altitude. We found evidence for an alternative energy converting system to gain a supplementary energy source. The energy converting system, bacteriorhodopsin, halorhodopsin and HTH domain proteins, were also found in comparison it to all other sequenced strains in the genus *Natrinema* and they mostly share this energy-producing pathway.

More intensive study and data-mining need to be considered in genomes of the genus *Natrinema* or another halophilic archaeon. Then, we might find some reasons for these ancient archaeon to have so much vitality and prosperity in extreme environment on planet Earth.

## Abbreviations

ANI: Average Nucleotide Identity
BBH: Bi-directional Best Hit
KAAS: KEGG Automatic Annotation Server
LPA: Ligation-mediated PCR Amplification
Plsm: Plasmid
